# Impact of dual temperature profile in dilute acid hydrolysis of spruce for ethanol production

**DOI:** 10.1186/1754-6834-3-15

**Published:** 2010-07-01

**Authors:** Peter Bösch, Ola Wallberg, Elisabeth Joelsson, Mats Galbe, Guido Zacchi

**Affiliations:** 1 Institute of Chemical Engineering, Vienna University of Technology, Getreidemarkt 9/E1662, A-1060 Vienna, Austria; 2 Department of Chemical Engineering, Lund University, P.O. Box 124, SE-221 00 Lund, Sweden

## Abstract

**Background:**

The two-step dilute acid hydrolysis (DAH) of softwood is costly in energy demands and capital costs. However, it has the advantage that hydrolysis and subsequent removal of hemicellulose-derived sugars can be carried out under conditions of low severity, resulting in a reduction in the level of sugar degradation products during the more severe subsequent steps of cellulose hydrolysis. In this paper, we discuss a single-step DAH method that incorporates a temperature profile at two levels. This profile should simulate the two-step process while removing its major disadvantage, that is, the washing step between the runs, which leads to increased energy demand.

**Results:**

The experiments were conducted in a reactor with a controlled temperature profile. The total dry matter content of the hydrolysate was up to 21.1% w/w, corresponding to a content of 15.5% w/w of water insoluble solids. The highest measured glucose yield, (18.3 g glucose per 100 g dry raw material), was obtained after DAH cycles of 3 min at 209°C and 6 min at 211°C with 1% H_2_SO_4_, which resulted in a total of 26.3 g solubilized C6 sugars per 100 g dry raw material. To estimate the remaining sugar potential, enzymatic hydrolysis (EH) of the solid fraction was also performed. EH of the solid residue increased the total level of solubilized C6 sugars to a maximum of 35.5 g per 100 g dry raw material when DAH was performed as described above (3 min at 210°C and 2 min at 211°C with 1% H_2_SO_4_).

**Conclusion:**

The dual-temperature DAH method did not yield decisively better results than the single-temperature, one-step DAH. When we compared the results with those of earlier studies, the hydrolysis performance was better than with the one-step DAH but not as well as that of the two-step, single-temperature DAH. Additional enzymatic hydrolysis resulted in lower levels of solubilized sugars compared with other studies on one-step DAH and two-step DAH followed by enzymatic hydrolysis. A two-step steam pretreatment with EH gave rise to a considerably higher sugar yield in this study.

## Background

The depletion of oil reserves and the effects of oil-based energy on the global climate have provided an incentive to search for alternative sources of energy. In particular the transport sector is dependent on oil-derived products. Ethanol is a potential substitute of gasoline as fuel for vehicles. It is currently derived from starch-rich substrates such as maize corn and plants with high sugar contents, such as sugarcane [1,2].

Another potential source of ethanol is lignocellulose [3,4]. Currently, however, lignocellulose cannot be directly fermented to ethanol. Before fermentation, a process is required to break down the lignocellulose into sugar monomers. Several methods are known to facilitate the degradation of the lignocellulose complex, including dilute acid hydrolysis (DAH), AFEX, pH control or lime methods [5-8]. Such pretreatment processes are followed by enzymatic hydrolysis (EH) to increase the total sugar yield. Acid hydrolysis processes are efficient in sugar polymer degradation as no EH is required before fermentation; however, the formation of inhibitors may be substantial, causing sugar losses. The overall cost of converting biomass to ethanol is significantly influenced by the costs of raw materials and of enzymes [9], and it is therefore crucial to select a pretreatment method that is appropriate for the raw material. In other words, the treatment should yield a product that is suitable for EH and that contains low levels of sugar degradation products, as these will inhibit the fermentation process [10]. In addition to carbohydrates, lignocellulose also contains lignin. To make the best use of the raw material, components other than the carbohydrates need to be utilized. One alternative is to produce ethanol from the sugars by fermentation and to use the residues, including lignin, as pellets or for the generation of electricity [9].

The raw material considered for this study was spruce, a type of softwood. Pretreatment of softwood has previously been investigated using methods such as impregnation with acid catalysts, (for example, SO_2 _and H_2_SO_4_) before steam pretreatment, and also DAH [11,12]. A two-step DAH method using H_2_SO_4 _and a two-step pretreatment method of an acid-impregnated lignocellulosic substrate followed by EH have been thoroughly explored [13-16]. The advantage of the two-step process was an increase in the hemicellulose yield, although the glucose yield remained the same [17].

The two-step DAH method comprises an acid hydrolysis step under conditions of low severity, which hydrolyzes the hemicellulose fraction. The solid material can then be washed and subjected to a second AH step, performed under conditions of greater severity, with the aim of degrading the cellulose. Washing the material between the steps removes the hemicellulose sugars and reduces the level of sugar-derived inhibitors, such as hydroxymethylfurfuraldehyde (HMF) and furfural, which are formed under more severe conditions [18]. This washing has to be conducted either at high pressure (1-2.5 MPa), which poses a serious engineering challenge, or at ambient pressure, which has the drawback of increased steam utilization, due to the material having to be reheated to the higher temperature. Before the second hydrolysis step, the material may also need to be impregnated again. A study by Monavari *et al*. suggests that removing the liquid by pressing between the two steps without washing the material leads to the best results for sugar yields [16].

In this study, we addressed the drawbacks of two-step processes by introducing a new reactor design with improved controllability, which permitted temperature profiles to be used during DAH. The two-step DAH was simulated by a one-step process that involved raising the temperature during the hydrolysis; the first period was conducted at a lower temperature to hydrolyze the hemicellulose, then the temperature was increased, but with a reduced residence time. The aim was to obtain a high hydrolysis yield of cellulose without generating inhibitors that were detrimental to the fermentation [10].

The primary objective of the study was to determine the efficiency of DAH using a dual temperature profile in a novel reactor setup. This was to be compared with single-level (regular hydrolysis) experiments so as to point out the effect of a more advanced temperature control; small reactors tend to suffer from temperature overshooting. Because the temperature behavior was of particular interest, a series of tests was conducted with an alternative temperature control strategy. Also, the EH of the slurry after DAH was investigated to estimate the potential increase in sugar yield that was obtainable when using EH as compared with DAH alone.

## Materials and methods

### Raw material

Fresh, chipped (20 mm long and approximately 5 mm thick) spruce (*Picea abies*) was kindly provided by a local sawmill (Widtsköfle Sågverk AB, Everöd, Sweden). The wood chips were milled and sieved to yield a suitable substrate for the acid hydrolysis (chips of 2.2 to 10 mm in size). This size reduction was necessary because of the diameter of the reactor's inlet valve and had a negligible effect on the pretreatment outcome [19]. The composition of the milled material is given in Table 1. The dry matter (DM) content of the sieved material was 41.7% w/w. The material was stored at 4°C in plastic bags until it was subjected to DAH.

**Table 1:** Composition of the raw material

Component	% w/w of DM
Glucan	45.1
Galactan	2.8
Mannan	11.8
Total C6 polymers	59.7
Xylan	5.2
Arabinan	1.1
Total C5 polymers	6.3
Acid-insoluble lignin	28.5
Acid-soluble lignin	3.8
Total lignin	32.5
Ash	0.2
Total components analyzed	98.6

### DAH

**Figure 1:**
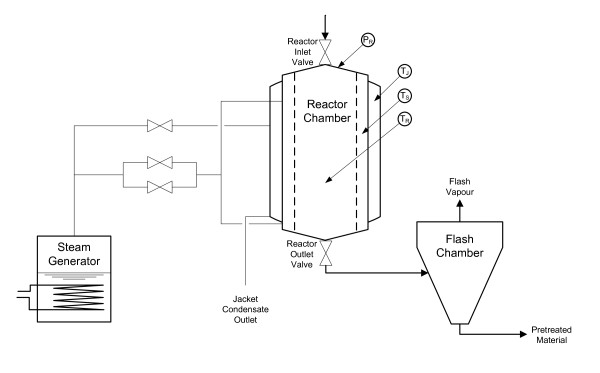
**Hydrolysis equipment**.

The DAH process is shown in Figure 1. The reactor was supplied with steam of the desired temperature by a steam generator. The steam pressure was reduced by a valve to the desired pressure corresponding to a particular temperature (190-220°C). The reactor was top-loaded with the substrate (spruce) and after closing the reactor inlet valve, the wood chips were subjected to direct steam until the temperature set-point for the reaction was reached. After the reaction time, the reactor outlet valve was opened and the material emptied - under the pressure from the reactor - into the flash vessel, where excess steam flashed off from the total slurry (TS). The acid-hydrolyzed material was then collected for further analysis.

To conduct the experiments, a new 4 liter reactor was used. A cylindrical metal mesh insert allowed improved steam distribution throughout the reactor. Compared with their larger counterparts, small pretreatment reactors, such as this one, have the disadvantage of an undesirably high surface to volume ratio, resulting in an increased consumption of steam required to compensate for heat losses, which further dilutes the slurry. This renders the reactor and the experimental results generally less valuable for scaling up to larger volumes. To address this issue, a heating jacket was used to maintain the surface temperature at the same level as the reactor temperature.

The control system comprised a controller made up of a processor unit (PM851K01) and an automation system (Industrial^IT ^800×A 5.0; both ABB, Malmö, Sweden). Three thermocouples measured the temperature in the chamber (T_R_), the space between the chamber wall and the mesh (T_S_), and the jacket (T_J_). The pressure in the reactor was monitored by the pressure sensor P_R _(Figure 1). The heating cycle was initiated by heating the jacket, followed by heating the chamber. After achieving the desired chamber temperature, the steam inlet valve was closed, and steam was introduced through a proportional-integral-derivative (PID) regulated control valve to maintain a constant temperature.

Before DAH was carried out, the spruce was impregnated for a minimum of 30 min with sulfuric acid to yield a concentration of 1% w/w based on the water content of the material. Each shot consisted of 350 g DM spruce, with shots duplicated to compensate for possible variations between them, before the flash vessel was emptied. The pretreatment conditions are summarized in Table 2. As can be seen, the temperature varied between 190 and 220°C, and the residence time for the total DAH reaction was between 3-15 minutes. The combined severity factor for these conditions ranged from 2.70 to 3.93 (see below).

**Table 2:** Hydrolysis conditions

Sample	t_1_, min	T_R1_, °C	t_2_, min	T_R2_, °C	CS	Sample	t_1_, min	T_R1_, °C	t_2_, min	T_R2_, °C	CS
1	3	199			2.70	26^b^	5	209	3	212	3.45
2	5	192			2.73	27^a^	5	210	2	220	3.50
3	5	193			2.74	28^b^	3	209	6	211	3.51
4	5	193			2.76	29^b^	5	206	6	210	3.54
5	5	194			2.77	30^b^	5	206	6	211	3.56
6	5	196			2.82	31	5	208	3	220	3.56
7	3	204			2.85	32	5	208	3	220	3.56
8	7	194			2.93	33^b^	5	205	8	210	3.62
9^a^	5	200			2.95	34	5	206	4	220	3.62
10	5	201			2.97	35^b^	5	207	8	210	3.63
11	5	201			2.98	36	5	209	4	220	3.64
12^a^	5	202			3.02	37^b^	5	206	10	211	3.71
13	5	203			3.03	38	5	203	6	220	3.72
14^a^	7	202			3.17	39	5	207	6	220	3.75
15	5	198	2	210	3.18	40	5	210	6	220	3.77
16^a, b^	3	210	2	211	3.26	41	5	206	8	220	3.84
17	5	203	3	211	3.35	42	5	208	8	220	3.85
18^b^	5	208	2	213	3.37	43	5	207	10	220	3.92
19^b^	3	208	4	211	3.37	44^a^	5	208	10	220	3.93
20^a, b^	5	208	2	213	3.38	45^a, c^	5	191	3	220	3.41
21	7	204	2	211	3.39	46^c^	5	200	3	220	3.47
22^a^	5	203	4	210	3.40	47^a, c^	5	191	4	220	3.51
23^b^	7	206	2	211	3.43	48^a, c^	5	202	4	220	3.58
24^b^	5	206	4	211	3.45	49^c^	5	191	6	220	3.67
25	5	206	2	221	3.45	50^a, c^	5	208	10	210	3.71

^a^Enzymatically hydrolyzed.

^b^Allocated to single-level pretreatment.

^c^Corresponds to changed reactor settings.

After a full series of experiments was completed and analysed, the influence of a modified control strategy for the reactor heating period was investigated. Instead of a heating pattern that tolerated a certain level of overshooting, the new regimen was set to reach the final reaction temperature after 30 seconds by slowly increasing the temperature.

### Analyses

Analysis of the raw material was performed in duplicate on samples of dried and milled spruce chips. The composition of the material was determined according to the National Renewable Energy Laboratory (NREL) Laboratory Analytical Procedures (LAP) [20]. After the acid hydrolysis, a sample of the slurry (about 30 g) was dried for 24 h at 105°C to determine the DM. The water-insoluble solids content (WIS) and the sugar and inhibitor concentrations in the slurry were also determined. The WIS was measured by stirring 100 g of slurry in 1 L of hot water, filtering off the liquid, and then rinsing with excess water, followed by determination of the dry matter content. Samples from EH were withdrawn at regular time intervals (0, 24, 48 and 72 h), and the sugar and the inhibitor concentrations were measured using a high-performance liquid chromatography system (Shimadzu Scientific Instruments, Columbia, MD, USA) equipped with a refractive index detector (RID-10A; Shimadzu Scientific Instruments). Before analysis, the samples were filtered through 0.2 μm disposable filters to remove particulates. The sugars (glucose, mannose, arabinose, galactose and xylose) were separated in a resin-based column (Aminex HPX-87P; Bio-Rad, Hercules, CA, USA) at 85°C with water as the eluent at a flow rate of 0.6 mL/min. The inhibitors (HMF, furfural, acetic acid, levulinic acid and formic acid) were analyzed using an Aminex HPX-87H column (Bio-Rad, Hercules, CA) at 65°C, 0.5 mL/min with 0.005 M H_2_SO_4 _as the eluent.

### EH

The slurry from the acid hydrolysis was subjected to EH to investigate the remaining sugar potential. An enzyme mixture of Celluclast 1.5L (58 filter paper units (FPU)/g and 17 IU/g of β-glucosidase) and Novozyme 188 (376 IU/g of β-glucosidase), both kindly donated by Novozymes A/S (Bagsværd, Denmark), was used. A water-insoluble solids (WIS) concentration of 2% w/w was chosen to avoid end-product inhibition in the determination of the potential sugar yield. A total of 10 g of WIS, 2.60 g of Celluclast and 0.46 g of Novozym were diluted in 0.1 mol/L of NaAc buffer to a total mass of 500 g. Hydrolysis was performed at 40°C and pH 4.8 for 72 h. The experiments were conducted in duplicate.

### Severity factor

Incorporating the parameters 'time' and 'temperature' [21], the severity factor R is defined as(1)

where t_1 _(min) is the residence time at the reaction temperature and T_R1 _(°C) is the temperature in the reactor. The function is extended for acid conditions to(2)

yielding the combined severity factor (CS) by subtracting the pH value based on the set acid concentration before the reaction.

To have the same convenient form for comparing the results of one-step pretreatments at different consecutive temperature levels (T_R _(°C)) and durations (t_i _(min)), the severity factor was extended to(3)

## Results and Discussion

### Reactor startup

Before the DAH experiments were conducted, preliminary testing of the reactor was required to establish the operation procedure. Part of this commissioning work involved determining the influence of the jacket heating, which should compensate for the disproportionate heat loss of small reactors. In theory, a higher temperature in the jacket than in the reactor should reduce heat loss and therefore the steam consumption in the reactor, yielding a higher dry matter content after the reaction. Experimental shots with a temperature difference (T_J_-T_R_) of up to 30°C were conducted. The obtained experimental data (not shown) led to the conclusion that the jacket temperature should be equal to the reactor temperature (T_J _= T_R_).

Although the reactor was fully insulated thermally, certain heat sinks could not be avoided. In particular, the reactor inlet and outlet valves and the steam inlet valves contributed to the total energy loss during the acid hydrolysis. Two valves controlled the influx of steam into the reaction chamber; the first one was fully opened to rapidly heat the spruce to the desired temperature and then closed, and the second valve, which was had finer control, was subsequently used to maintain a constant temperature. To reach the second temperature level, the first (larger) steam valve was re-opened. Any condensate that formed in the proximity of this valve was then transferred into the chamber. This contributed to the lower DM of the two-level experiments compared with their single-level counterparts.

The reactor heating was completed in a matter of seconds. The response of the temperature sensor was too slow to control the initial heating, which resulted in a temperature overshoot of 5-10°C. This problem was addressed by using the pressure sensor in the chamber in conjunction with a correlation between the saturation pressure of water and the temperature for the initial phase, which resulted in good accuracy, thereby eliminating most of the temperature overshooting. This control strategy was then substituted for the previous one.

### DAH

**Figure 2:**
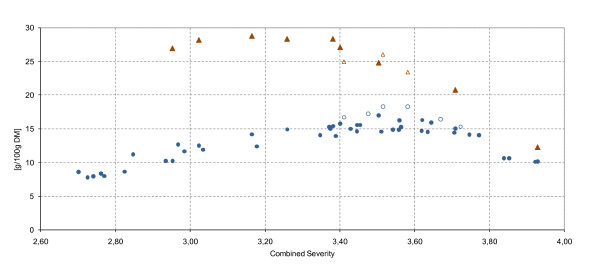
**Impact of modified reactor control strategy on sugar yield**. Solubilized glucose (g per 100 g). Filled black circle (open circle), glucose after dilute acid hydrolysis (DAH); filled black triangle (open triangle), total level of glucose after enzymatic hydrolysis as a function of the combined severity. Open symbols correspond to a modified reactor control; theoretical maximum 50.1 g glucose/100 g dry matter (DM).

In total, 50 conditions were investigated (Table 2). Samples marked with 'c' are singled out in the plots (open symbols in Figure 2) because they were conducted to test an additional reactor control strategy. They are therefore discussed separately later.

**Figure 3:**
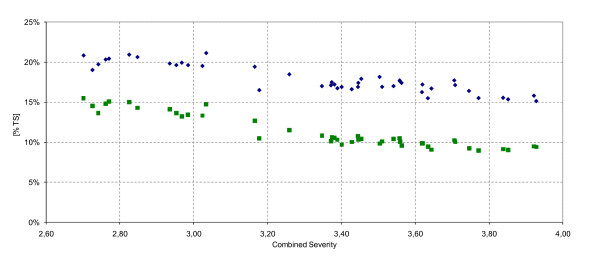
**Impact of combined severity on dry matter and water-insoluble solids**. Filled black diamond, dry matter as percentage of the total level of slurry; filled black square, water-insoluble solids content (WIS) as percentage of the total level of slurry as a function of the combined severity.

The initial DM of the raw material was 41.7% w/w. The maximum DM content after hydrolysis, assuming a dilution by the steam condensate used to heat up the material, was estimated to be 34.7% (a heat capacity of spruce of 3.18 kJ/(kg K) at 100°C was assumed [22]). This did not take into account the loss of flash vapor. Figure 3 shows the resultant DM and WIS after DAH for the various investigated conditions. When the CS was increased, the DM content of the total slurry dropped from 21.1% to 15.1% w/w. Samples from DAH without the second temperature step (1-14) were slightly drier, because of the issues with the steam inlet valve mentioned above.

Experiments were also performed to evaluate the steam consumption of the empty reactor (data not shown). Because the jacket was already at the operating temperature when the reactor heating was started, no heat losses and therefore no condensation of steam was expected at the reactor walls. However, a major source of heat loss occurred at the material inlet and outlet valves, and the condensate formed diluted the material. Based on the experimental results with the empty reactor, the level of additional liquid could be calculated. As an example, without heat losses by the inlet and outlet valves, the DM would hypothetically increase from 20% to 25% w/w for sample 10 (201°C for 5 min; Table 2).

With increasing CS, the level of WIS decreased from 15.5 to 9.0% w/w. This corresponded to a WIS recovery level of 64.1 to 39.0% w/w. When harsher treatment conditions were applied, more material was hydrolyzed to yield soluble compounds, due to an increased sugar polymer to monomer ratio at higher temperatures. Again, the samples subjected to dual temperatures were found to become more dilute as a result of the additional condensate from the steam inlet valve.

**Figure 4:**
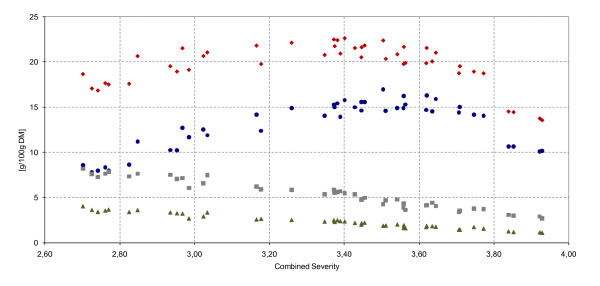
**Solubilized sugars after weak acid hydrolysis (g per 100 g dry matter (DM))**. Filled black triangle, xylose (max 5.9 g/100 g DM); filled black square, mannose (maximum 13.1 g/100 g DM); filled black circle, glucose (maximum 50.1 g per 100 g DM); filled black diamond, total C6 sugars after DAH (maximum. 66.3 g per 100 g DM).

The total level of C6 sugars (recalculated to monomer sugars from Table 1) available in the raw material leveled at 66.3 g per 100 g DM, with glucose constituting 50.1 g per 100 g DM. The highest yield of glucose obtained after acid hydrolysis (that is, 17.0 g glucose per 100 g raw material (33.9% of theoretical value)), was found for combined severities between 3.37 to 3.71 (Figure 4). Below this CS range, the reaction rate from polymeric sugar to monosaccharide was too low, whereas above it, the degradation of the glucose to HMF outperformed the formation of sugars. Hemicellulose was hydrolyzed at a lower severity than cellulose. At a CS of 2.70, the yield of xylose was 4.02 g/100 g and that of mannose was 8.18 g/100 g. In the specified CS band, xylose and mannose yields were increasingly degraded to furfural and HMF, respectively, thus reducing the yield to 1.12 and 2.67 g/100 g at a CS of 3.93, respectively.

The highest yields of hexose sugar (glucose, mannose, galactose) were found for a CS levels between 2.85 and 3.64. The maximum obtained value of solubilized C6 sugars was 22.6 g per 100 g raw material at a CS of 3.40 (34.1% of theoretical value). Owing to the increase degradation of the hemicellulose-derived C6 sugars with increasing severity, the maximum yield of overall C6 sugars was produced at lower severity than that of the maximum glucose yields.

Nguyen *et al*. conducted two-stage DAH at 200°C for 5 min with 0.4% H_2_SO_4_; 215°C for 3 min at 0.4% H_2_SO_4_; 190°C for 3 min at 0.7% H_2_SO_4_; and 215°C for 3 min at 0.4% H_2_SO_4 _[13]. The reported glucose yields were 16.7 and 16.0 g per 100 g raw material, which was only slightly lower than the maximum 17 g per 100 g raw material we found in this study. However, the glucan share of the raw material (approx 70%white fir (*Abies concolor*) and 30% Ponderosa pine (*Pinus ponderosa*)) was only 33.9% w/w, whereas in our study the substrate had a glucan share 45.1% w/w which definitely affected the glucose yield that is, the solubilized monomeric glucose with respect to the dry raw material). Nguyen *et al*. found 44.8% and 42.6%, in contrast to the 33.9% we measured.

**Figure 5:**
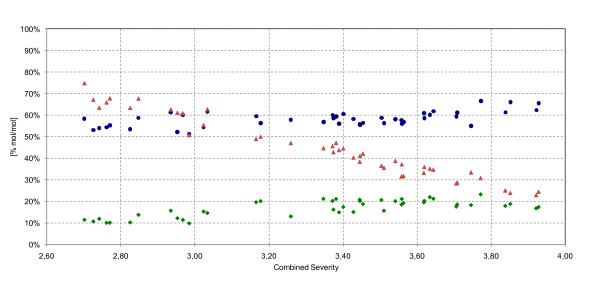
**C5 sugars and degradation products of the total slurry as a percentage of the total C5 sugars in the raw material (mol/mol)**. Filled black triangle, xylose + arabinose; filled black triangle, furfural; filled black circle, acetic acid as a function of the combined severity.

**Figure 6:**
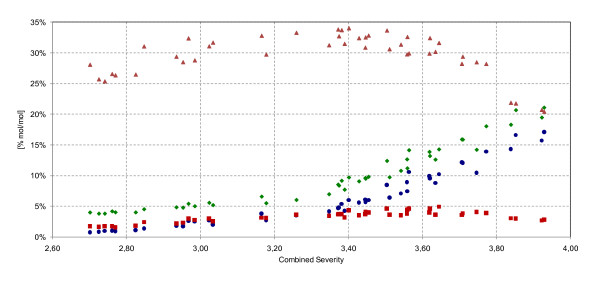
**C6 sugars and degradation products in the total slurry as a percentage of the total C6 sugars in the raw material (% mol/mol)**. Filled black triangle, glucose + mannose + galactose; filled black diamond, formic acid; filled black circle, levulinic acid; filled black square, hydroxymethylfurfuraldehyde (HMF).

The formation of sugar-derived degradation products increased significantly when the severity was increased: the pentoses xylose and arabinose were degraded to furfural, which was then further oxidized to formic acid [23]. The level of C5 sugars in the pretreated material declined rapidly, as shown in Figure 5. Whereas 75% mol/mol C5 sugars in the raw material could be recovered at a CS of 2.70, only 23% mol/mol were recovered at a CS of 3.93. Compared with the C5 sugars, the formation of furfural increased as the severity was raised. The low level of furfural in comparison to that of C5 sugars can be explained by its volatility; the furfural evaporated by flashing (that is, by release of pressure) and by its subsequent degradation to formic acid. The ratio of acetic acid increased only slightly, thus confirming the high degree of hemicellulose degradation under the specified conditions. Hexoses were degraded to HMF (ratio 1:1), which was then further degraded to formic acid and levulinic acid (ratio 1:1:1) [23]. The profile of the HMF formation (Figure 6) followed the same trend as the monosaccharide formation, but was slightly shifted along the CS axis. The peak of the HMF formation was found at severities of 3.4 to 3.6. This was believed to result from the rate of degradation of HMF to acids becoming higher than the formation of HMF as glucose formation was limited by the reaction of cellulose to glucose. Mannose also contributed to the formation of HMF. The formic acid formation followed the same trend as that of levulinic acid but was shifted by 3% mol/mol due to the contribution of the furfural degradation, which was evident even at the lowest of the investigated severity levels. The acid formation showed a steep increase in reaction rate at CS 3.40 and this coincided with the highest hexose yield and a leveling-off at the maximum of the HMF concentration.

Söderström *et al*. reported that the highest inhibitor concentration for a two-step dilute acid pretreatment of softwood (spruce) occurred at 180°C for 10 min at 0.5% H_2_SO_4 _and 220°C for 2 min at 1% H_2_SO_4 _[15]. The reported inhibitor levels were 3.9 g/L HMF and 1.3 g/L furfural. These values were considerably lower than those obtained in our study, in which about 5.5 g/L HMF and 2.5 g/L furfural were detected. However, in the investigation by Söderström *et al*., the substrate was washed after the first step, which reduced the level of C5 and C6 sugars (originating from the hemicellulose fraction) that were available for degradation in the second step. By contrast, in our two-level DAH procedure, washing between the steps was omitted.

### Effect of a dual temperature profile

Samples 1-14 were conducted as single-temperature experiments and sample 15-50 as dual temperature experiments. For some of the latter experiments, the temperatures in the two steps differed by less than 5°C and these were thus allocated to the single-level runs (see Table 2, indicated by 'b').

In terms of combined severity, a range from 2.70 to 3.93 was covered. Within this range, samples of single/dual-temperature DAH had overlapping severities, ranging from 3.26 to 3.71 CS, thereby spanning the most relevant conditions in terms of C6 sugar yields. The analysis of the data allowed the following conclusions:

• The full spectrum of CS from 2.70 to 3.93 showed no discontinuity, favoring neither single-level nor two-level pretreatment but following a comprehensible trend. This was true for hexose, pentose and the degradation product analyses, and for EH.

• In the overlapping range (3.26 to 3.71), the data points of single/dual-temperature DAH had a random scatter pattern. No trend with regard to yields, favoring one process or the other, could be established.

Based on these results, both pretreatment methods were equivalent for this type of setup in terms of sugar yields and degradation product formation. Furthermore, no influence on the C6 sugar yield after EH was evident. It should be noted that the scattering of the data points was very low for this type of experiment, which supports the conclusions drawn from the analysis. The time required to conduct the pretreatment was well below 10 min. By contrast, the experiments conducted by Söderström *et al. *[15] required 12 min just for pretreating the material. Nevertheless, the time for to heat, wash and drain the reactor and to handle the material should be taken into account. The consolidation into a two-level process results in lower capital costs.

### EH of acid-hydrolyzed material

**Figure 7:**
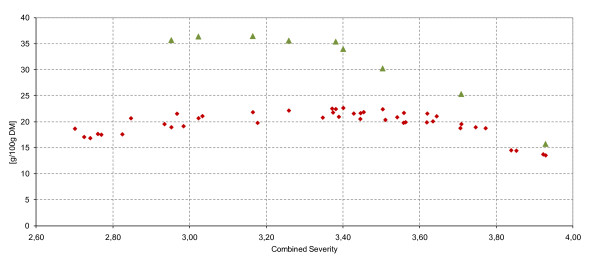
**Solubilized sugars (g per 100 g)**. Filled black diamond, total level of C6 sugars after dilute acid hydrolysis (DAH); filled black triangle, total level of C6 sugars after enzymatic hydrolysis (EH) as a function of the combined severity; theoretical maximum 66.3 g C6/100 g dry matter (DM).

**Figure 8:**
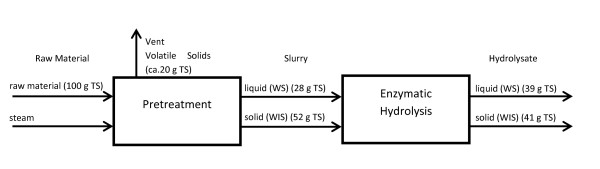
**Flow diagram of the sugar solubilization by pretreatment at a combined severity factor (CS) of 3.17 followed by enzymatic hydrolysis (EH)**.

EH of the total slurry was performed for selected samples to evaluate which sugar yield was potentially obtainable. The highest level of solubilized hexoses, (that is, 36.4 g per 100 g DM (55% of theoretical value)), was found for DAH at a CS of 3.17 (corresponding to 1% H_2_SO_4_, for 7 min at 202°C). Figure 7 shows a plateau of 35 g per 100 g DM for a CS of 2.95 to 3.38 with an assumed peak at 3.02 to 3.17. At a CS of 3.40 and higher, a rapid decline in hexoses was measured. The maximum yield of glucose after EH was 28.8 g per 100 g DM at a CS of 3.17 (57.5% of theoretical value), from which 14.6 g per 100 g DM was released by EH. The best-case scenario is shown in detail by Table 3 with a supporting process flow diagram (Figure 8).

**Table 3:** Example of sugar solubilization (yield is g per 100 g unless otherwise stated) with raw material, liquid fraction of the slurry after pretreatment, and liquid fraction after EH (hydrolysate) with a combined severity factor of 3.17 (corresponding to 1% H2_S_O_4 _for 7 min at 202°C).

	Raw material	Slurry	Hydrolysate
Glucose	50.1	14.2	28.8
Galactose	3.2	1.4	1.6
Mannose	13.1	6.2	6.4
Cellubiose	1	1.0	1.0
Xylose	5.8	2.6	2.8
Arabinose	1.3	0.9	N/A

Overall yield hexoses	66.3	21.8	36.7
Yield hexoses (g/g total C6), %	-	33	55

Liquid	100	28	39^4^
Solids (WIS)	-	52	41

Total solids, %	41.7 (37.7^2^)	19.4 (12.7^3^)	-

^1^Cellubiose is accounted for in glucose fraction.

^2^After impregnation with H2SO4,

^3^WIS.

^4^Small error with sugar balance due to sampling issue with greatly diluted specimen.

A previous study on a two-step dilute-acid pretreatment of softwood followed by EH was conducted by Söderström *et al. *[15]. The glucose yield after two pretreatments was around 19 g per 100 g DM (180°C for 10 min at 0.5% H_2_SO_4 _and 200°C for 2 min at 2% H_2_SO_4_). The reported combined (that is, pretreatment steps 1 and 2 plus EH) glucose yield of 36 g per 100 g DM (75% of theoretical value) was thus considerably higher than the results we obtained. Both the pretreatment and the EH performed better than the best case observed in our study.

A similar study by Nguyen *et al. *on a single-stage dilute-acid pretreatment of softwood forest thinnings followed by EH resulted in a final glucose yield after EH of 33.3 g per 100 g DM (75% of the theoretical value), with 13.3 g per 100 g DM (30% of theoretical value) available after pretreatment [12]. The pretreatment conditions were 215°C for 1.66 min at 0.65% H_2_SO_4_. Although the results of the pretreatment were slightly inferior to ours with regard to glucose yield, EH performed much better, with an increase of about 20.0 g per 100 g DM, resulting in a larger amount of total available glucose.

Both the studies conducted by Söderström *et al. *and Nguyen *et al. *relied on the material being washed before EH. By contrast, we performed EH on the whole slurry without washing, which might partly explain the low yield in the hydrolysis step, which might be a result of possible inhibition of the enzymes [18]. Another explanation could be the high level of sugar-degradation products as reported above, which diminished the level of cellulose available for EH.

The optimum CS range in our study was 2.85 to 3.64 for DAH, and 3.02 to 3.17 for the pretreatment and subsequent EH in terms of solubilized hexoses. The overlapping CS ranges open up opportunities for facility design. First a DAH process should be established, and in cases where additional EH is found to be more economical, the process can be extended with slight modifications of the earlier DAH conditions; we believe that this should increase the hexose sugar yield 34% to 55% of the total C6 sugar available.

### Modified reactor control strategy

Samples hydrolyzed with a reactor control strategy that requires more time to reach the set reaction temperature, but avoids overshooting (Table 2; Figure 2 open symbols), concurred with the general trends of DM and WIS. The DM was slightly increased compared with specimens from previous experiments conducted under equivalent conditions of severity. By contrast, the WIS, was exactly in line with the previous results. An increased level of DM at constant WIS indicated a higher level of solubilized sugars. This was further confirmed by the results for the monosaccharides glucose, mannose and xylose. The highest glucose yield (18.3 g per 100 g DM), was obtained at a CS of 3.58 (Figure 2)., corresponding to an increase of 1.3 g glucose per 100 g DM (7.6%). The highest level of solubilized C6 sugars occurred at a CS of 3.51 with 26.3 g per 100 g DM (39.6% of theoretical value).

Three samples were enzymatically hydrolyzed with the modified setup. Although the initial sugar concentration was high, the increase due to EH was low, indicating a high conversion rate of cellulose to glucose during DAH. For example, at a CS of 3.51, approximately 29.5% more hexose sugars were available after EH, whereas at a CS of 3.50, with the previous reactor configuration, the hexose sugar yield due to EH increased by 35%.

## Conclusion

DAH with either single or dual temperature levels was investigated at varying temperatures and reaction times. The analysis of the experimental data led to the conclusion that the difference in performance between single- and dual-temperature DAH was minimal. Based on the defined CS both types of temperature profiles resulted in equivalent performances with respect to inhibitor levels and sugar yields. To estimate the remaining sugar potential, EH was conducted on selected samples. Again, single- and dual-temperature profiles gave comparable outcomes.

The obtained results were compared with counterparts from DAH, two-step DAH and a two-step pretreatment followed by EH. The glucose yield was found to be higher than in DAH but lower than in the two-step DAH and the two-step pretreatment followed by EH. EH was much more efficient in DAH, two-step DAH and two-step pretreatment processes compared with single- or dual-temperature process. A possible explanation for this discrepancy could be a higher degree of inhibitor formation compared with the two-step pretreatment or EH of the native slurry used instead of the washed material used in the other studies.

The procedure described in this paper requires fewer utilities (mainly process steam) and less equipment due to a shorter residence time and no washing step as demanded by the two-step pretreatment. However, the performance was inferior to that found in a study conducted by Söderström, who found higher yields and lower levels of inhibitors.

When increasing the temperature from one level to the next, the mesh inside the chamber had a beneficial effect, facilitating a faster heat distribution because the already partly degraded material was not clumped together at the bottom. With increasing reactor size, there is less heat loss per kg of material because the volume to surface ratio is improved. This should be considered when scaling up the process, because a drier hydrolysate has different, but not necessarily better, characteristics.

Changing the reactor heating settings resulted in higher yields of glucose and total hexoses after DAH, thus increasing the robustness range of the process. Additional EH had reduced potential because less cellulose was available in the slurry. This indicates a promising pathway for future improvements. Further work is needed to confirm the enhancements on sugar yields after DAH.

Although the established data did not clearly show a means of process improvement for the pretreatment with two temperature levels, a different temperature profile (ramp, exponential increase), changed chamber settings, or an alternative substrate might have a positive effect on the process performance, enabling it to approach the performance obtained by the two-step pretreatment.

## Competing interests

The authors declare that they have no competing interests

## Authors' contributions

PB carried out the DAH, and EH experiments, performed the analysis and drafted the manuscript. OW conceived the study, and designed the reactor and control system. EJ carried out the EH experiments and analysis. MG designed the reactor and control system, and performed analysis. GZ conceived the study. All authors took part in planning the study, checking the results and writing the manuscript.

## References

[B1] Wheals AE Basso LC Alves DMG Amorim HV Fuel ethanol after 25 years Trends Biotechnol 1999 17 482 487 10.1016/S0167-7799(99)01384-0 10557161

[B2] Farrell A Plevin RJ Turner BT Jones DA O'Hare M Kammen DM Ethanol can contribute to energy and environmental goals Science 2006 311 506 508 10.1126/science.1121416 16439656

[B3] Hahn-Hägerdal B Galbe M Gorwa-Grauslund MF Liden G Zacchi G Bio-ethanol - the fuel of tomorrow from the residues of today Trends Biotechnol 2006 24 549 556 10.1016/j.tibtech.2006.10.004 17050014

[B4] Balat M Balat H Öz C Progress in bioethanol processing Prog Energy Combust 2008 34 551 573 10.1016/j.pecs.2007.11.001

[B5] Duff SJB Murray WD Bioconversion of forest products industry waste cellulosics to fuel ethanol: a review Bioresour Technol 1996 55 1 33 10.1016/0960-8524(95)00122-0

[B6] Galbe M Zacchi G Pretreatment of lignocellulosic materials for efficient bioethanol production Adv Biochem Eng Biotechnol 2007 108 41 65 17646946 10.1007/10_2007_070

[B7] Sun Y Cheng J Hydrolysis of lignocellulosic materials for ethanol production: a review Bioresour Technol 2002 83 1 11 10.1016/S0960-8524(01)00212-7 12058826

[B8] Yang B Wyman CE Pretreatment: the key to unlocking low-cost cellulosic ethanol Biofuels Bioprod Biorefin 2008 2 26 40 10.1002/bbb.49

[B9] Sassner P Galbe M Zacchi G Techno-economic evaluation of bioethanol production from three different lignocellulosic materials Biomass Bioenerg 2008 32 422 430 10.1016/j.biombioe.2007.10.014

[B10] Larsson S Palmqvist E Hahn-Hägerdal B Tengborg C Stenberg K Zacchi G Nilvebrant N-O The generation of fermentation inhibitors during dilute acid hydrolysis of softwood Enzyme Microb Technol 1999 3-4 151 159 10.1016/S0141-0229(98)00101-X

[B11] Galbe M Zacchi G A review of the production of ethanol from softwood Appl Microbiol Biotechnol 2002 59 618 628 10.1007/s00253-002-1058-9 12226717

[B12] Nguyen Q Tucker M Keller F Eddy F Two-stage dilute-acid pretreatment of softwoods Appl Biochem Biotechnol 2000 84 561 576 10.1385/ABAB:84-86:1-9:561 10849819

[B13] Nguyen Q Tucker M Keller F Beaty D Conners K Eddy F Dilute acid hydrolysis of softwoods Appl Biochem Biotechnol 1999 77 133 142 10.1385/ABAB:77:1-3:133

[B14] Söderström J Pilcher L Galbe M Zacchi G Two-step steam pretreatment of softwood with SO_2 _impregnation for ethanol production Appl Biochem Biotechnol 2002 98 5 21 10.1385/ABAB:98-100:1-9:5 12018277

[B15] Söderström J Pilcher L Galbe M Zacchi G Two-step steam pretreatment of softwood by dilute H_2_SO_4 _impregnation for ethanol production Biomass Bioenerg 2003 24 475 486 10.1016/S0961-9534(02)00148-4

[B16] Monavari S Galbe M Zacchi G The influence of solid/liquid separation techniques on the sugar yield in two-step dilute acid hydrolysis of softwood followed by enzymatic hydrolysis Biotechnol Biofuels 2009 2 6 10.1186/1754-6834-2-6 19291286 PMC2661319

[B17] Söderström J Galbe M Zacchi G The effect of washing on yield in one- and two-step steam pretreatment of softwood for production of ethanol Biotechnol Progr 2004 3 744 749 10.1021/bp034353o 15176877

[B18] Tengborg C Galbe M Zacchi G Reduced inhibition of enzymatic hydrolysis of steam-pretreated softwood Enzyme Microb Technol 2001 28 835 844 10.1016/S0141-0229(01)00342-8 11397466

[B19] Ballesteros I Oliva J Navarro A Gonzales A Carrasco J Ballesteros M Effect of chip size on steam explosion pretreatment of softwood Appl Biochem Biotechnol 2000 84-86 97 110 10.1385/ABAB:84-86:1-9:97 10849782

[B20] Sluiter A Hames B Ruiz R Scarlata C Sluiter J Templeton D Crocker D Determination of structural carbohydrates and lignin in biomass NREL/TP-510-42618 Revised: Apr. 2008

[B21] Chum HL Johnson DK Black SK Overend RP Pretreatment - catalyst effects and the combined severity parameter Appl Biochem Biotechnol 1990 24/25 1 14 10.1007/BF02920229

[B22] Forest Products Laboratory Wood Handbook-Wood as an Engineering Material. General technical report FPL-GTR-113 1999 Madison, WI: US Department of Agriculturem, Forest Service, Forest Products Laboratory

[B23] Larsson S Ethanol from Lignocellulose - fermentation inhibitors, detoxification and genetic engineering of *Saccharomyces cerevisiae *for enhanced resistance PhD thesis 2000 Lund University, Department of Chemical Engineering

